# Prospective isolation of chondroprogenitors from human iPSCs based on cell surface markers identified using a CRISPR-Cas9-generated reporter

**DOI:** 10.1186/s13287-020-01597-8

**Published:** 2020-02-18

**Authors:** Amanda Dicks, Chia-Lung Wu, Nancy Steward, Shaunak S. Adkar, Charles A. Gersbach, Farshid Guilak

**Affiliations:** 1 Department of Orthopaedic Surgery, Washington University, St. Louis, MO 63110 USA; 2 Shriners Hospitals for Children – St. Louis, St. Louis, MO 63110 USA; 3 Department of Biomedical Engineering, Washington University, St. Louis, MO 63110 USA; 4 Center of Regenerative Medicine, Washington University, St. Louis, MO 63110 USA; 5 Department of Cell Biology, Duke University Medical Center, Durham, NC 27710 USA; 6 Department of Biomedical Engineering, Duke University, Durham, NC 27710 USA

**Keywords:** hiPSC, Chondroprogenitor, Chondrocyte, Cartilage, Surface markers, Differentiation, Single-cell RNA sequencing

## Abstract

**Background:**

Articular cartilage shows little or no capacity for intrinsic repair, generating a critical need of regenerative therapies for joint injuries and diseases such as osteoarthritis. Human-induced pluripotent stem cells (hiPSCs) offer a promising cell source for cartilage tissue engineering and in vitro human disease modeling; however, off-target differentiation remains a challenge during hiPSC chondrogenesis. Therefore, the objective of this study was to identify cell surface markers that define the true chondroprogenitor population and use these markers to purify iPSCs as a means of improving the homogeneity and efficiency of hiPSC chondrogenic differentiation.

**Methods:**

We used a CRISPR-Cas9-edited *COL2A1-GFP* knock-in reporter hiPSC line, coupled with a surface marker screen, to identify a novel chondroprogenitor population. Single-cell RNA sequencing was then used to analyze the distinct clusters within the population. An unpaired *t* test with Welch’s correction or an unpaired Kolmogorov-Smirnov test was performed with significance reported at a 95% confidence interval.

**Results:**

Chondroprogenitors expressing CD146, CD166, and PDGFRβ, but not CD45, made up an average of 16.8% of the total population. Under chondrogenic culture conditions, these triple-positive chondroprogenitor cells demonstrated decreased heterogeneity as measured by single-cell RNA sequencing with fewer clusters (9 clusters in unsorted vs. 6 in sorted populations) closer together. Additionally, there was more robust and homogenous matrix production (unsorted: 1.5 ng/ng vs. sorted: 19.9 ng/ng sGAG/DNA; *p* < 0.001) with significantly higher chondrogenic gene expression (i.e., *SOX9*, *COL2A1*, *ACAN*; *p* < 0.05).

**Conclusions:**

Overall, this study has identified a unique hiPSC-derived subpopulation of chondroprogenitors that are CD146^+^/CD166^+^/PDGFRβ^+^/CD45^−^ and exhibit high chondrogenic potential, providing a purified cell source for cartilage tissue engineering or disease modeling studies.

## Background

Articular cartilage is the load-bearing tissue that lines the ends of long bones in diarthrodial joints, serving to resist compression and provide a nearly frictionless surface during joint loading and movement [1, 2]. The extracellular matrix of cartilage is comprised primarily of type II collagen and proteoglycans, which are synthesized by the main residing cell type, chondrocytes [3, 4]. However, because it is aneural and avascular, cartilage shows little or no capacity for intrinsic repair [4]. Traumatic injury and a chronic inflammatory state lead to irreversible degeneration of the tissue, driving diseases such as osteoarthritis (OA) [5, 6]. Current treatments only target disease symptoms, creating a great demand for tissue-engineered cartilage as a system for disease modeling, drug testing, and tissue replacement.

Human-induced pluripotent stem cells (hiPSCs) offer a promising source for cartilage tissue engineering and in vitro disease modeling [7] as they have virtually unlimited expansion capacity, can be genetically modified, and can avoid many of the ethical considerations associated with embryonic stem cells [8, 9]. Despite reports of several chondrogenic differentiation protocols for pluripotent stem cells [10–15], incomplete differentiation and cell heterogeneity remain as the major obstacles for iPSC chondrogenesis [16, 17]. This challenge has been addressed in other stem and progenitor cell types by prospectively isolating cells that exhibit chondrogenic lineage commitment using surface marker expression. For example, previous studies have identified chondroprogenitors within adult articular cartilage that can be isolated using fibronectin adhesion assays since progenitors express integrins α5 and β1 [18, 19]. Additionally, mesenchymal progenitor cells, which express CD105, CD166 (ALCAM), and CD146 (MCAM), have been reported to have a high chondrogenic potential [19–21]. Adult multipotent cells, such as the bone marrow-derived mesenchymal stem cells (MSCs) or adipose-derived stem cells (ASCs), exhibit chondrogenic potential and have been used extensively for cartilage tissue engineering. They are often characterized by a range of cell surface marker expression, including CD105, CD73, CD90, CD271, CD146, Stro-1, and SSEA-4 [22]. In an effort to identify a more developmentally relevant progenitor population, self-renewing human skeletal stem cells characterized by CD164^+^, CD73^−^, and CD146^−^ showed chondrogenic differentiation when implanted in a mouse renal capsule [23]. In another study, limb bud cells expressing CD73 and BMPR1β while having low to no expression of CD166, CD146, and CD44 were proposed to be the earliest cartilage committed cells (prechondrocytes) in human embryonic development [24]. However, surface marker characteristics of hiPSC-derived chondroprogenitors or chondrocytes remain to be identified.

Previously, our lab used green fluorescent protein (GFP) reporter systems to track the expression of collagen type II alpha 1 chain (*COL2A1*) in mouse [25] and human [26] iPSCs, allowing for the prospective isolation and purification of *COL2A1*-*GFP*+ chondrogenic cells during the differentiation process. Despite the fact that this approach significantly enhanced homogeneity of iPSC chondrogenesis [26], genome editing is required to create a reporter line, hindering potential clinical translation. In this regard, the identification of cell surface markers that are directly representative of this *COL2A1*-positive population could greatly enhance the prospective isolation and purification of chondroprogenitors, without requiring genetic modifications to the cell line.

In this study, we used a *COL2A1*-GFP knock-in reporter hiPSC line as a tool to identify cell surface markers that are highly co-expressed with *COL2A1* to test the hypothesis that this subpopulation of chondroprogenitor cells will show increased purity and chondrogenic capacity. Single-cell RNA sequencing (scRNA-seq) was then used to investigate the gene expression profile of this population and to identify subsets within it. Matrix production, cell morphology, and gene expression were measured to evaluate chondrogenic ability of unsorted and sorted chondroprogenitor cells. This chondroprogenitor population appears to represent an intermediate step in the developmental pathway of in vitro hiPSC chondrogenesis in which off-target differentiation also occurs. The identification of surface markers to purify this population of chondroprogenitor cells via sorting will enhance the efficiency of hiPSC chondrogenic differentiation for use in tissue engineering, in vitro disease modeling, and drug testing.

## Methods

Materials and methods are briefly summarized. A detailed description is provided in supplemental information.

### hiPSC lines and culture

Two hiPSC lines were used in the current study: RVR *COL2A1-*GFP knock-in line (RVR) and BJFF.6 line (BJFF). Both lines were maintained on vitronectin-coated plates (VTN-N; Fisher Scientific, USA, A14700) with daily medium changes. Cells were passaged at approximately 90% confluency and induced into mesodermal differentiation at 40% confluency.

### Mesodermal differentiation

hiPSCs were induced into mesodermal differentiation in monolayer according to the previously published protocol [26]. In brief, cells were fed daily with various cocktails of growth factors and small molecules driving lineage differentiation (anterior primitive streak, paraxial mesoderm, early somite, sclerotome, and chondroprogenitor) in differentiation medium. Upon differentiation into the chondroprogenitor stage, cells were dissociated and were used for cell sorting and chondrogenic differentiation as appropriate.

### Fluorescence-activated cell sorting (FACS)

Chondroprogenitor cells were resuspended in FACS Buffer (phosphate-buffered saline without calcium and magnesium (PBS^−/−^; Gibco, USA, 14-190-250) with 1% fetal bovine serum (FBS) and 1% penicillin/streptomyocin/fungizone (P/S/F; Gibco, USA, 15-240-062) and stained with various antibodies that are conventionally considered markers for mesenchymal progenitor cells (Supplemental Table S1). Cells were then sorted by an Aria-II FACS machine.

### 10X chromium platform scRNA-seq

Cells were thawed at 37 °C and resuspended in PBS^−/−^ with 0.01% bovine serum albumin (BSA; Invitrogen, USA, AM2616) at a concentration of 2000 cells/μl. Cell suspensions were submitted to the Genome Technology Access Center (GTAC sequencing core) at Washington University in St. Louis for library preparation and sequencing. In brief, 10,000 cells per sample were loaded on a Chromium Controller (10X Genomics, USA) for single capture. Detailed methods for quality control and processing for scRNA-sea data are described in supplemental information.

### Expansion of chondroprogenitor cells

Sorted and unsorted chondroprogenitor cells were plated on non-coated flasks and cultured in MEM alpha media (Gibco, USA, 12571048) with 1% penicillin/streptomycin (P/S; Gibco, USA, 15070063), 50 μg/ml l-ascorbic acid 2-phosphate (ascorbate; Sigma-Aldrich, USA, A4544), and 10 ng/ml basic fibroblast growth factor (bFGF; R&D Systems, USA, 233FB001MGC). Cells were fed every 3 days until 80–90% confluency prior to further expansion or chondrogenesis. Chondroprogenitor cells were passaged up to four times.

### Chondrogenic differentiation

Sorted, unsorted, and expanded chondroprogenitor cells (after 12 days of mesodermal differentiation) were resuspended at 3 × 10^5^ cells/mL in chondrogenic medium supplemented with 10 ng/ml human transforming growth factor-beta 3 (TGF-β3; R&D Systems, USA, 24-3B3-200CF). Chondrogenic pellets were cultured at 37 °C for 28 days. The medium was changed every 3–4 days.

### Histology and immunohistochemistry

After 28 days of chondrogenic differentiation, pellets were fixed and sectioned at 8 μm. Slides were either stained with safranin-O and hematoxylin for glycosaminoglycans evaluation or labeled against various collagen antibodies including COL1A1, COL2A1, COL6A1, and COL10A1.

### Biochemical analysis

Chondrogenic pellets (28 days of chondrogenesis) were digested at 65 °C overnight in a papain solution. The PicoGreen (Invitrogen, USA, P7589) and 1,9-dimethylmethylene blue (Sigma-Aldrich, USA, 341088), with chondroitin-4-sulfate (Sigma-Aldrich, USA, C9819) as a standard, assays were used according to the protocols to quantify DNA and sGAG, respectively.

### Gene expression

Chondroprogenitor cells were lysed and day 28 pellets were homogenized. Gene expression was analyzed using the ΔΔC_T_ method relative to undifferentiated hiPSCs with the reference gene TATA-box-binding protein (*TBP*) [27]. An alternative method to analyze gene expression normalized the C_T_ value of the gene of interest to that of the *TBP* for the same sample. Sequences of primers can be found in the Supplemental Table S2.

### Statistical analysis

Quantification of surface marker expression was performed 8 separate times with technical replicates of *n* = 3–4 for each experiment. Biochemical analysis and RT-qPCR were performed on the pellets collected from two independent sorting experiments (*n* = 3–4 samples per group per experiment). Gene expression and sGAG/DNA data were tested for normality using the Shapiro-Wilk test. An unpaired *t* test with Welch’s correction was then performed assuming a Gaussian distribution. If data was not normal, an unpaired Kolmogorov-Smirnov test was performed. All calculations were performed using GraphPad Prism (GraphPad Software; version 8.0). Two-tailed *p* values were calculated and reported at a 95% confidence interval.

## Results

### *COL2A1*-positive chondroprogenitor cells express PDGFRβ, CD146, and CD166

**Fig. 1:**
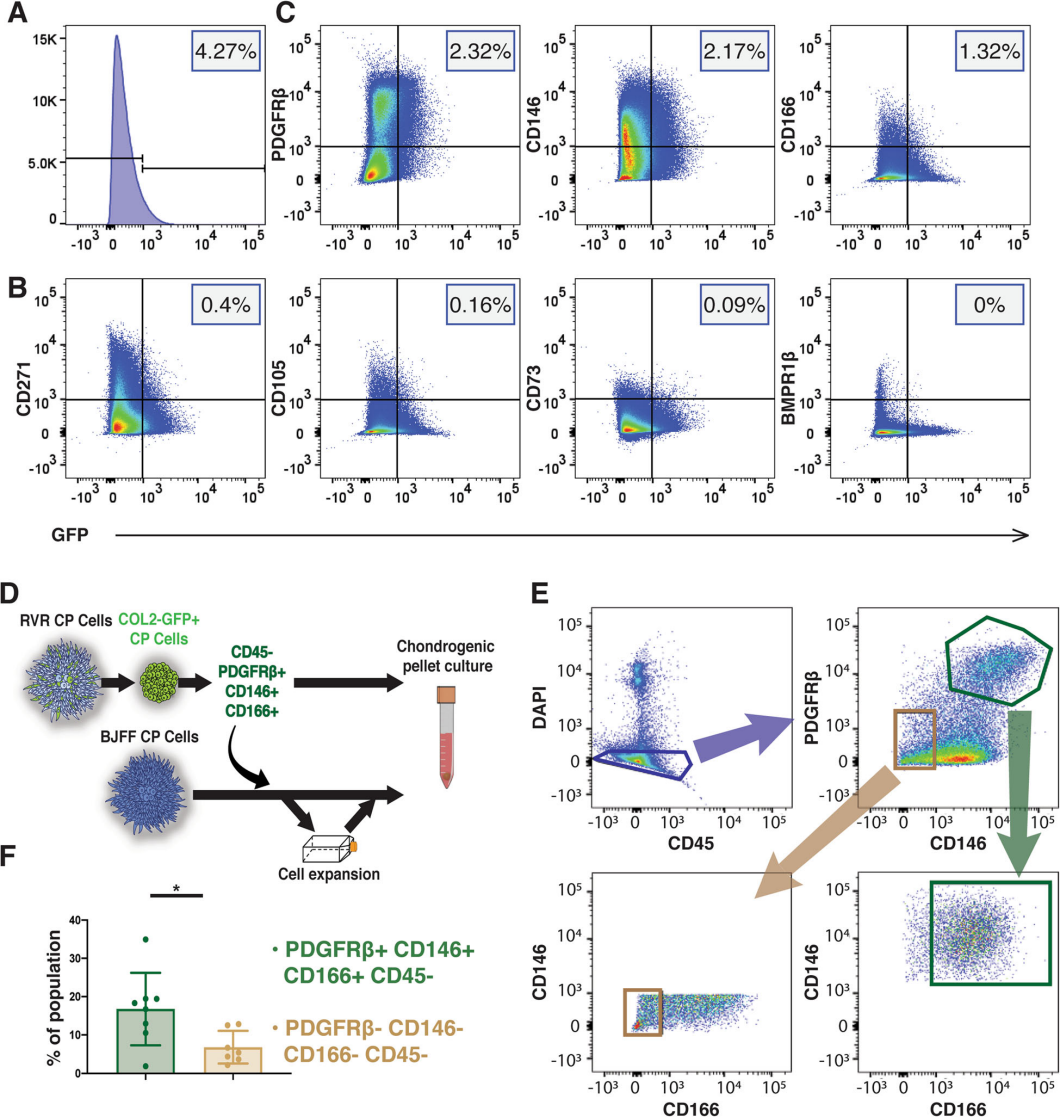
Surface marker analysis and sorting strategy to identify progenitors with robust chondrogenic potential from heterogenous chondroprogenitor (CP) cells. **a** Flow cytometry showed approximately 4.27% of cells expressed *COL2A1-GFP*. **b**, **c** Chondroprogenitors were labeled for various surface markers and analyzed for co-expression with *COL2A1-GFP*. **b** Most *COL2A1-GFP*^+^ cells did not express CD271, CD105, CD73, and BMPR1β. **c** PDGFRβ, CD146, and CD166 were co-expressed with *COL2A1*-*GFP*. **d** A schematic representing the experimental design. The RVR cell line with the *COL2A1*-*GFP* reporter was differentiated into chondroprogenitor cells. Surface marker analysis indicated that PDGFRβ, CD146, and CD166 expression were highly co-expressed with *COL2A1* but not CD45. **e** Cells expressing these desired markers were sorted from wildtype BJFF chondroprogenitor cells. To evaluate the chondrogenic potential of the sorted cells, pellets from the sorted cells were either made immediately post-sorting or formed after in vitro expansion. **f** A higher percentage of the total cell population (~ 16.8%) was triple positive for the desired markers compared to the population not expressing any of these markers. *p < 0.05. Data represented as mean ± SEM. *n* = 7–8 independent experiments. See also Figure S1

*COL2A1*-GFP reporter hiPSCs were differentiated into chondroprogenitor cells along the mesodermal lineage for 12 days as previously described [26]. After the 12 days of differentiation, flow cytometric analysis showed that, on average, 4.27% of the population expressed *COL2A1* based on *GFP* expression (Fig. 1a). The *COL2A1-*positive cells were assumed to be chondroprogenitors with a unique surface marker profile. The cells were labeled for surface markers commonly associated with MSCs and/or chondroprogenitors in the developing limb bud: BMPR1β, CD73, CD105, CD146, CD166, CD271, and PDGFRβ [22–24]. Of the total population, less than 1% expressed *COL2A1* in addition to either CD271 (0.4%), CD105 (0.16%), CD73 (0.09%), or BMPR1β (0%) (Fig. 1b). Interestingly, 2.32%, 2.17%, or 1.32% of the total population co-expressed *COL2A1* with PDGFRβ, CD146, or CD166, respectively (Fig. 1c). Since these markers appear to be the most highly correlated with *COL2A1* expression of the previously identified as MSC and/or chondroprogenitor markers selected, cells were sorted based on the expression of these markers for this study. Sorting also removed cells expressing CD45 (< 15% of total cells) to eliminate any non-chondrogenic hematopoietic stem cells potentially derived during mesoderm differentiation (Fig. 1d).

### PDGFRβ-, CD146-, and CD166-enriched chondroprogenitor cells

The BJFF hiPSC line (wildtype without genome editing) was differentiated into chondroprogenitor cells accordingly (12 days in a monolayer). Cells, either directly underwent chondrogenic pellet culture, were expanded, were saved for scRNA-seq, or were labeled for the surface markers of interest (Fig. 1d). Fluorescence-activated cell sorting (FACS) was used to sort live chondroprogenitor cells negative for CD45 and positively expressing PDGFRβ and CD146, followed by expression of CD166 (Fig. 1e). Cells not expressing any of these surface markers were also analyzed as a negative control. Approximately 16.5% of the total chondroprogenitor cell population was triple positive for PDGFRβ, CD146, and CD166, which was significantly higher than the percentage of the cells (7.2% of the total cell population) that were triple-negative for these markers (Fig. 1f). As with unsorted cells, sorted cells were also collected and either pelleted for chondrogenesis, expanded, or saved for scRNA-seq, as described in Fig. 1d.

### scRNA-seq reveals that unsorted chondroprogenitor cells contained diverse cell populations

**Fig. 2:**
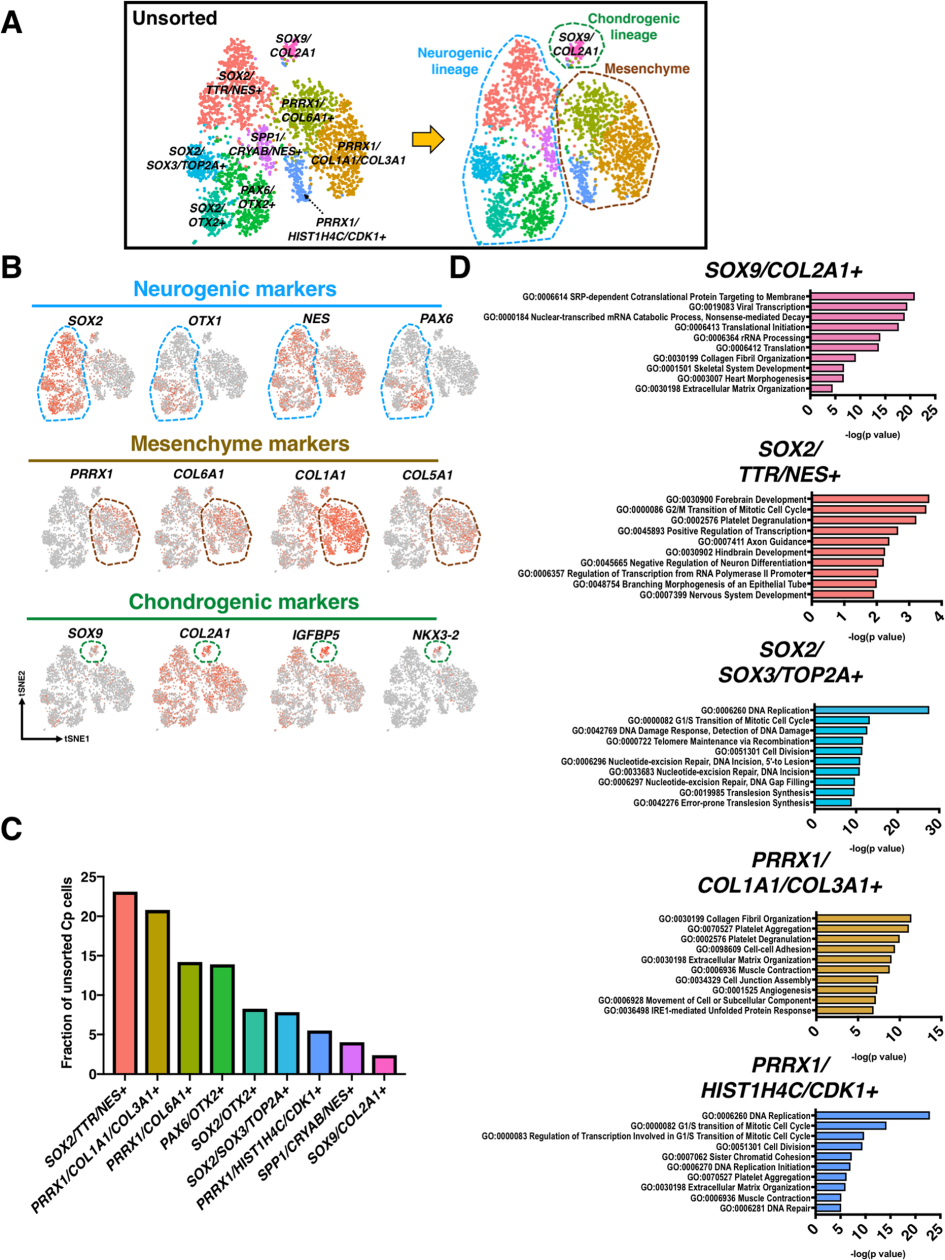
Cell populations and GO enrichment analysis of unsorted chondroprogenitor cells. **a** scRNA-seq identified unsorted chondroprogenitor cells contained at least 9 populations, which could be further categorized into 3 broad classes: neurogenic cells (blue dashed circle), chondrogenic cells (green dashed circle), and mesenchyme (brown dashed circle). **b** Expression of signature genes of each cell lineage. **c** GO terms analysis (biological process) of each unique population. **d** Percentage of total unsorted chondroprogenitor cells in each unique cell population. More than 20% of the unsorted chondroprogenitors were *SOX2/TTR/NES+* neurogenic cells, while only small number of unsorted cells expressed *SOX9* and *COL2A1*. See also Figure S1

We next used scRNA-seq to explore the cell diversity and genetic profiles of unsorted chondroprogenitor cells. At least 9 distinct cell populations (cell clusters) were observed in unsorted chondroprogenitor cells (Fig. 2a). Among these populations, 5 of them were enriched for a variety of neural cell markers such as *SOX2*, *OTX1*, *NES*, and *PAX6* (Fig. 2b), likely representing populations of the neurogenic lineage. Of these, *SOX2*, *OTX1*, and PAX6 expression were significantly downregulated with sorting according to RT-qPCR (Figure S2A). Furthermore, we found that 3 cell populations exhibited high expression levels of several mesenchyme markers including *PRRX1*, *COL1A1*, *COL5A1*, and *COL6A1* which were comparable between sorted and unsorted groups, while only a small cell population (2.3% of total cells) expressed chondrogenic markers such as *SOX9*, *COL2A1*, *IGFBP5*, and *NKX3-2* (Fig. 2b, c, S2B, and S2C). Using Gene Ontology (GO) enrichment analysis of the gene sets representing each cell cluster (Fig. 2d and S1A), we observed that cells expressing *SOX9* and *COL2A1* demonstrated gene sets enriched for protein translation and skeletal system development.

### scRNA-seq reveals that sorting enriched *SOX9/COL2A1+* cells

**Fig. 3:**
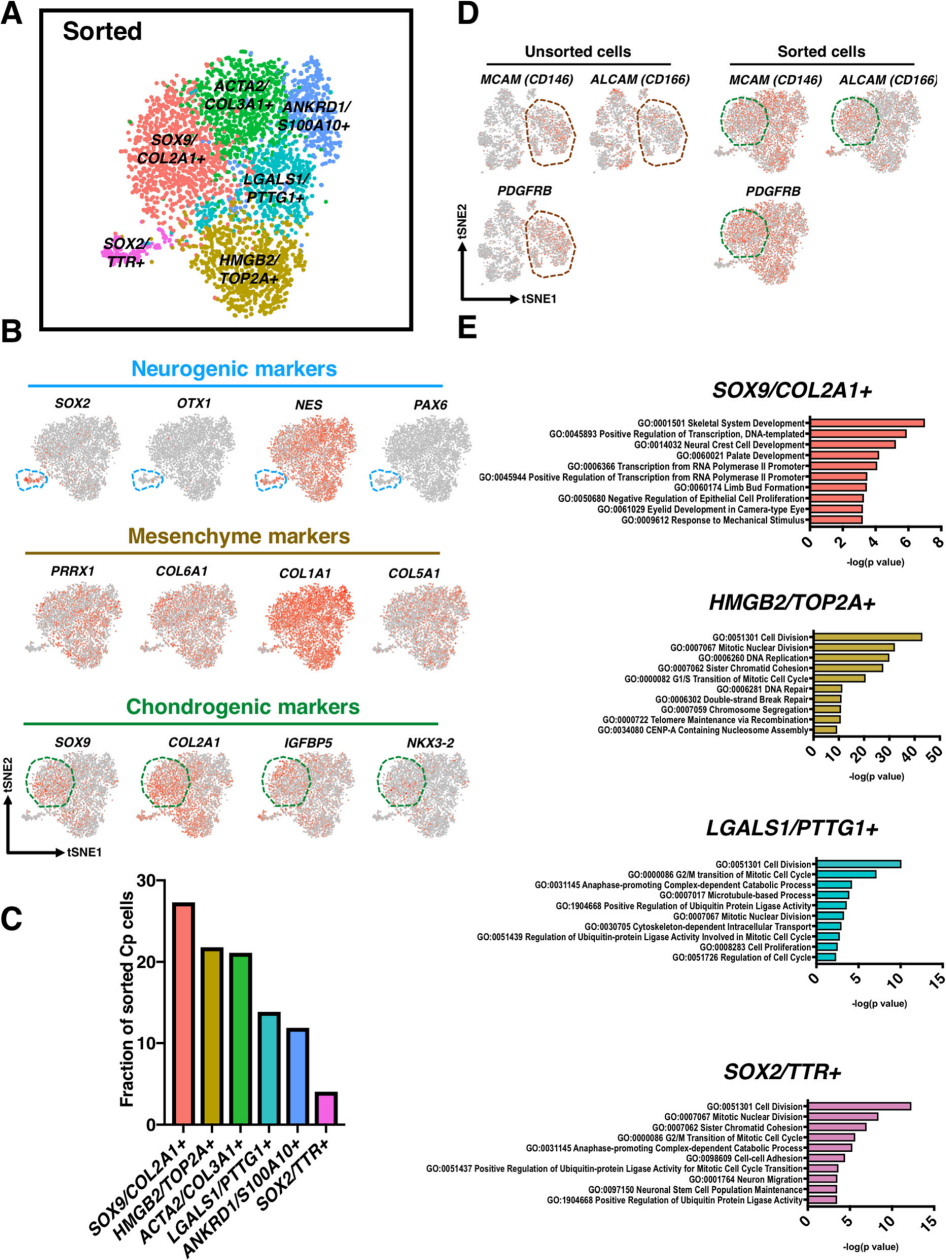
Cell populations and GO enrichment analysis of sorted chondroprogenitor cells. **a** scRNA-seq identified PDGFRβ^+^/CD146^+^/CD166^+^ cells contained at least 6 populations. **b** Expression of signature genes of each cell lineage. The sorted cells were enriched for mesenchymal and chondrogenic genes. **c** Percentage of total sorted chondroprogenitor cells in each unique cell population. Twenty-seven percent of the sorted were *SOX9/COL2A1.* Interestingly, a small percentage of cells (4% of total sorted cells) expressing *SOX2* and *NES* was still observed. **d** PDGFRβ^+^/CD146^+^/CD166^+^ sorted cells may belong to the mesenchymal population (brown dashed circle) in unsorted cells. The green dashed circle indicates the population that was positive for *SOX9* and *COL2A1.***e** GO terms analysis (biological process) showing skeletal system development was highlighted in *SOX9/COL2A1+* cells, while *HMGB2/TOP2A+* and *LGALS1/PTTG1+* cells were enriched in gene sets of cell division. See also Figure S1

scRNA-seq of sorted chondroprogenitor cells indicated that there were at least 6 cell populations consisting of PDGFRβ^+^/CD146^+^/CD166^+^ cells (Fig. 3a). Surprisingly, there was still a small percentage of cells (4% of total sorted cells) expressing *SOX2* and *NES*, despite the stringent sorting regime (Fig. 3b, c). We also observed that *SOX2*/*NES*^*+*^ cells exhibited a high expression of CD47, an integrin-associated protein [28] (Figure S1B). Nevertheless, sorting still significantly enriched cells positive for *SOX9* and *COL2A1* by > 11-fold (27% of total sorted cells vs. 2.3% of total unsorted cells). Interestingly, the overall gene expression of these chondrogenic genes was not increased. In fact, *COL2A1* was decreased with sorting when evaluated by RT-qPCR (Figure S2C). We observed that sorting slightly increased the percentage of the cells expressing ALCAM (CD166, 22.1% of the unsorted cells vs. 28.3% of the sorted cells). However, 9.9% of the total sorted cells were triple positive for *SOX9/COL2A1/ALCAM*, while only 0.8% of the total unsorted cells co-expressed these three genes. Interestingly, we also found that *ALCAM* was also expressed by both chondrogenic and neurogenic progenitors (e.g., 31.9% of *SOX9/COL2A1*^+^ cells and 44.9% of *SOX2/TTR*^+^ cells were positive for *ALCAM* in the sorted group), implying ALCAM alone may not be used as a sole marker for chondroprogenitor cells. Additionally, we also observed that gene expression levels of the sorting makers were enriched in the sorted population, with *ALCAM* highest in the *SOX9* and *COL2A1* cluster compared to the enrichment of all three in the unsorted mesenchyme population (Fig. 3d). Similarly, there was an enrichment of some previously reported pro-chondrogenic markers [18, 19, 21, 24] in the sorted chondroprogenitor population; specifically *ITGA5* and *ENG* (CD105) (Figure S3). Skeletal system development, as expected, emerged as a significant GO term in *SOX9/COL2A1+* cells, while *HMGB2/TOP2A+* and *LGALS1/PTTG1+* cells were enriched in gene sets of cell division (Fig. 3e and S1C).

### Canonical correlation analysis (CCA) demonstrates high enrichment of proliferative and mesenchymal genes in sorted chondroprogenitor cells

**Fig. 4:**
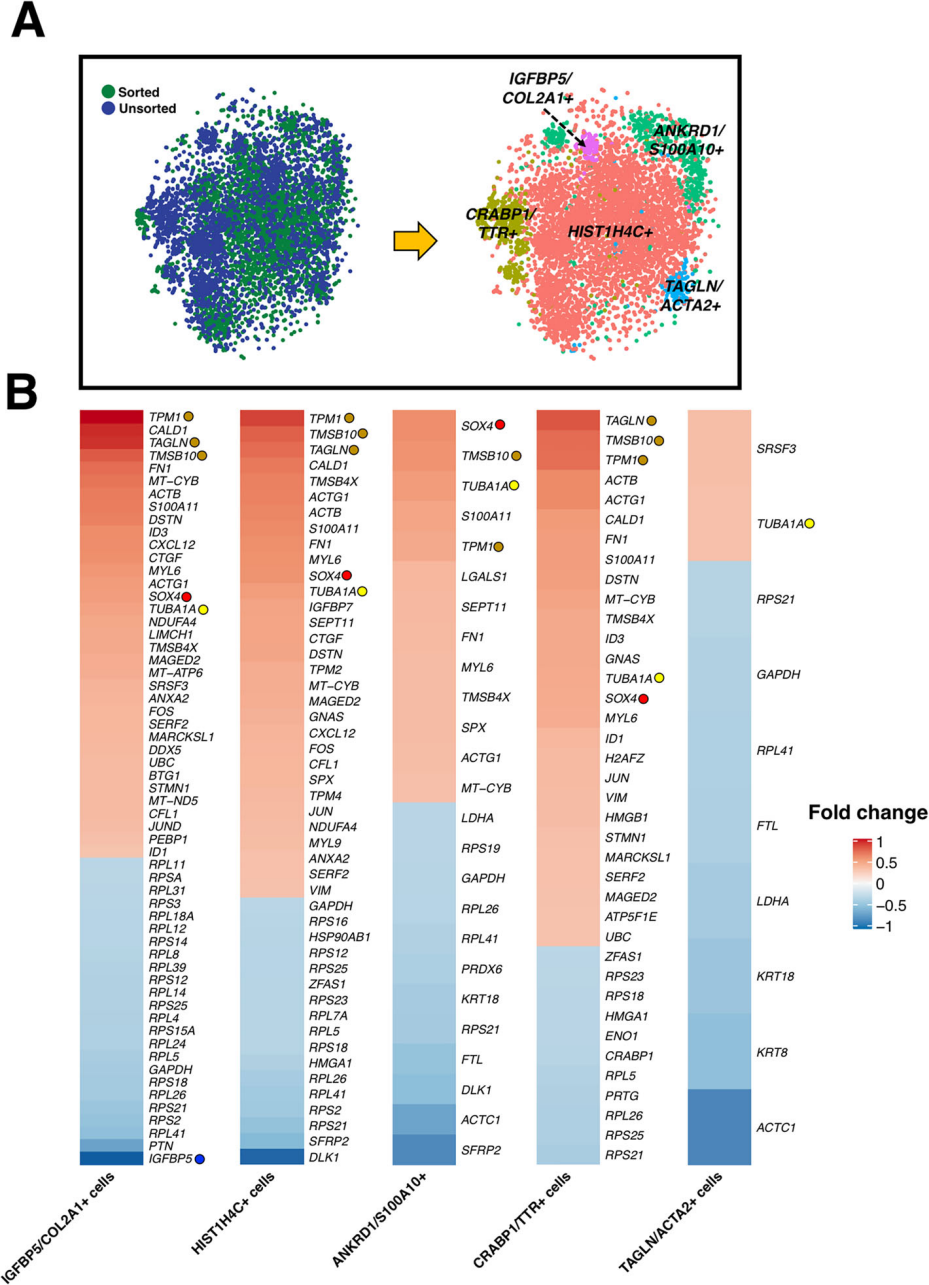
CCA for integrated analysis of sorted and unsorted scRNA-seq datasets. **a** Five major conserved populations were identified after CCA alignment of the sorted and unsorted chondroprogenitor cells. **b** DEG analysis indicated that sorted cells exhibited significantly upregulated expression of several mesenchymal genes including *TPM1*, *TAGLN*, and *TMSB10* (brown circle), which have been suggested to be essential in chondrogenesis. Proliferative markers including *SOX4* (red circle) and *TUBA1A* (yellow circle) were increased, but *IGFBP5* (blue circle) and several ribosomal genes were decreased in sorted cells

CCA, a machine-learning method that performs linear combinations of features across data sets that are maximally correlated, was used to integrate scRNA-seq datasets from sorted and unsorted cells [29]. Five major conserved populations were identified after CCA alignment of the sorted and unsorted chondroprogenitor cells (Fig. 4a). Among these populations, *HIST1H4C*^*+*^ cells accounted for the largest conserved population, while the *IGFBP5/COL2A1*^*+*^ cluster was the smallest. We next explored how sorting enriches or decreases the levels of gene expression within each individual population by analyzing differentially expressed genes (DEGs) (Fig. 4b). Within the *IGFBP5/COL2A1*^*+*^ population, sorted cells exhibited significantly upregulated expression of several mesenchymal genes including *TPM1*, *TAGLN*, and *TMSB10* (indicated by the brown circle), which have been suggested to be essential in chondrogenesis [30, 31]. Furthermore, within the *IGFBP5/COL2A1*^*+*^ population, sorted cells demonstrated significantly downregulated expression of *IGFBP5* (indicated by the blue circle), an important transcription factor inducing chondroprogenitor cells into the chondrogenic lineage [32].

### Sorting improved matrix production and homogeneity in cartilaginous pellets

**Fig. 5:**
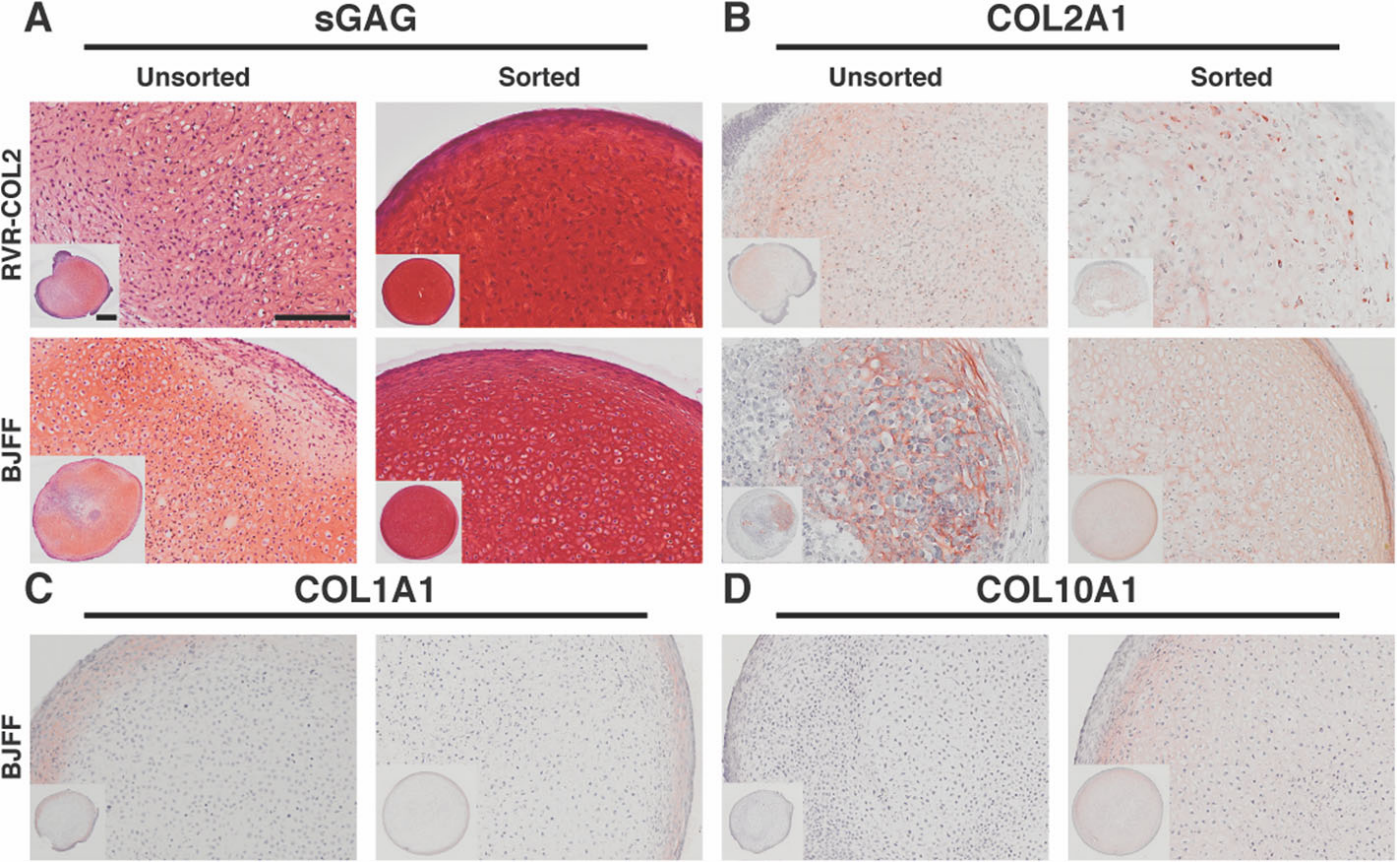
Histology and IHC for matrix proteins in RVR-COL2 and BJFF pellets. **a** Safranin-O staining for sGAG showing pellets derived from sorted chondroprogenitor cells had more robust staining and homogenous cell morphology compared to pellets derived from unsorted cells in both lines. **b** Labeling of COL2A1 showed similar results with an increase in COL2A1 in sorted pellets as opposed to unsorted which has isolated areas of staining. **c** There was little labeling of COL1A1 for both unsorted and sorted cell pellets. **d** Labeling for COL10A1 was increased with sorting. Scale bar = 200 μm. Inset scale bar = 400 μm. See also Figure S4 and S5

**Fig. 6:**
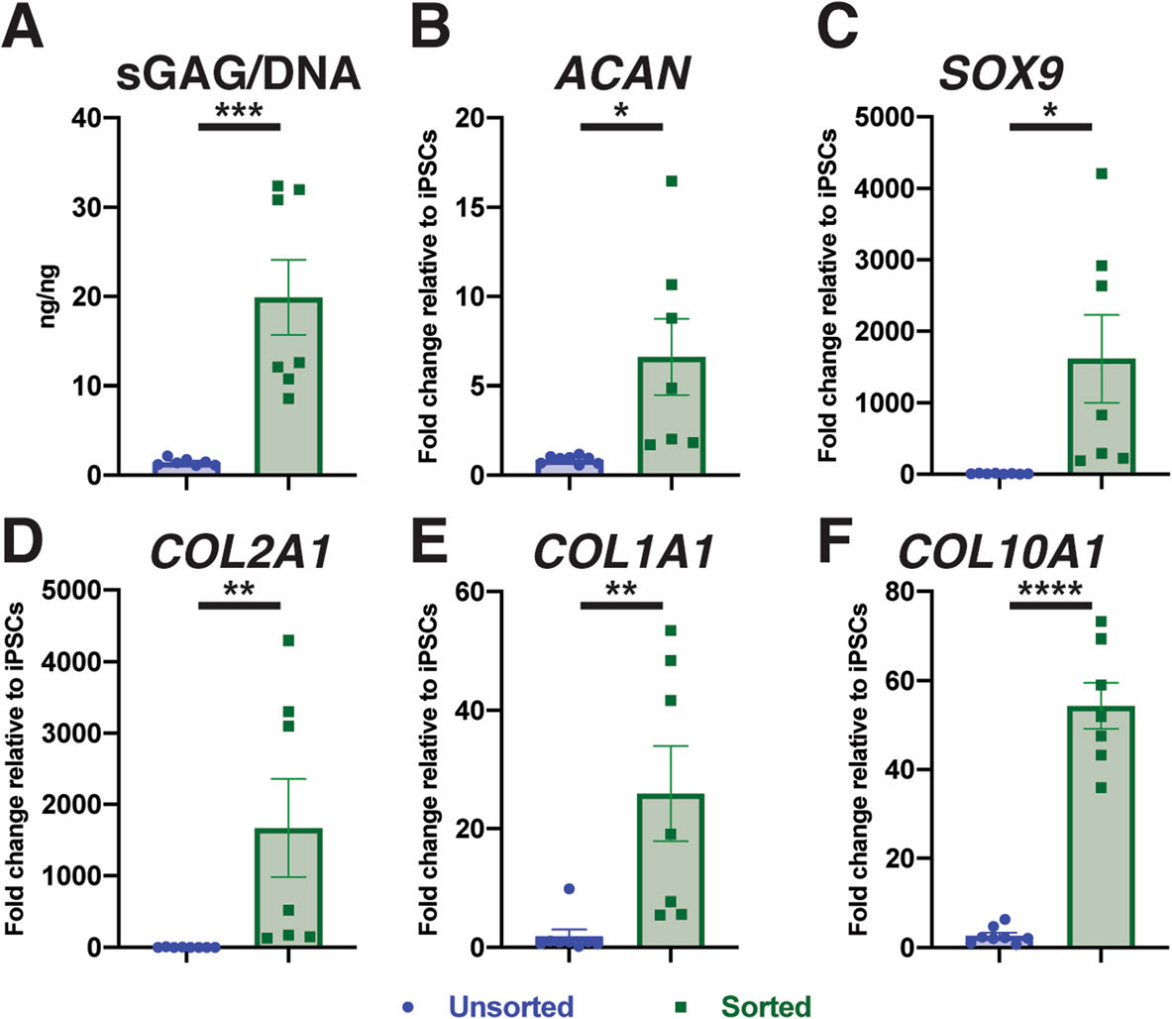
Quantitative analysis of matrix production and gene expression. **a** Sorting of chondroprogenitor cells prior to chondrogenesis significantly increased the sGAG/DNA ratio to approximately 20 ng/ng. **b**–**d** Expression of chondrogenic genes *ACAN*, *SOX9*, and *COL2A1* was significantly increased with sorting. **e**, **f** Sorting significantly unregulated fibrocartilage and bone matrix marker *COL1A1*, and hypertrophic cartilage marker *COL10A1*. Gene expression in reference to undifferentiated hiPSCs with housekeeping gene TBP. *p < 0.05, **p < 0.01, ***p < 0.001, and ****p < 0.0001. Data represented as mean ± SEM. *n* = 6–7 per group: 2 experimental replicates, 3–4 technical replicates (pellets). See also Figure S6

Sorted and unsorted cells from both the reporter and wildtype lines underwent chondrogenesis in pellet culture for 28 days. Pellets stained with safranin-O for sulfated glycosaminoglycans (sGAGs) showed that sorting increased matrix production as well as homogeneity of cell morphology (Fig. 5a and S4). Additionally, the layer of non-cartilaginous-like cells surrounding unsorted cell pellets was eliminated in the pellets derived from sorted cells. Biochemical analysis demonstrated that sorting significantly increased the ratio of sGAGs to DNA in pellets by almost 15-fold (unsorted: 1.5 ng/ng vs. sorted: 19.89 ng/ng, Fig. 6a). Similarly, there was an increase in production and homogeneity observed in sorted pellets labeled for COL2A1 (Fig. 5b). In addition, IHC labeling for COL1A1 showed a slight decrease at the perimeter of the pellet while the labeling for COL10A1 showed an increase in the respective matrix proteins with sorting (Fig. 5c, d). Additionally, pellets formed with sorted cells had more localized staining of COL6A1 around the cells as shown with IHC compared to the more diffused pattern observed with unsorted cells (Figure S5).

### Expression of cartilaginous genes was significantly higher in pellets derived from triple-positive chondroprogenitor cells

Gene expression in pellets derived from unsorted and triple-positive sorted chondroprogenitor cells was analyzed using RT-qPCR. Chondrogenic genes *SOX9* (unsorted 0.88-fold change vs. sorted 6.62 fold change), *ACAN* (unsorted 7.22-fold change vs. sorted 1614-fold change), and *COL2A1* (unsorted 0.68-fold change vs. sorted 1667-fold change) were significantly increased in sorted pellets (Fig. 6b–d). Additionally, *COL1A1* (unsorted 0.74-fold change vs. sorted 25.91-fold change) and *COL10A1* (unsorted 2.69-fold change vs. sorted 54.32-fold change) were significantly higher in sorted pellets compared to unsorted (Fig. 6e, f). Statistical significance was maintained for all genes when analyzed alternatively (Figure S6).

### Chondrogenic capacity was maintained through one passage of unsorted and sorted chondroprogenitor cells

Pellets derived from passage 1 (p1) sorted cells exhibited the most robust and homogenous safranin-O staining as compared to the pellets derived from sorted cells of later passages and to the pellets derived from unsorted cells of a similar passage (Figure S7). Pellets derived from p2-4 unsorted and sorted chondroprogenitor cells had comparable staining and cell morphology with decreased chondrogenic capacity (Figure S7).

## Discussion

Using a *COL2A1-*GFP reporter line, we identified a novel combination of surface markers (i.e., PDGFRβ^+^/CD146^+^/CD166^+^/CD45^−^) depicting a unique progenitor population with robust chondrogenic potential in hiPSC chondrogenesis. This finding was further confirmed by significantly increased cartilaginous matrix production of the prospectively isolated cells with these selected markers from a wildtype, non-edited hiPSC line. The results of scRNA-seq of sorted cells revealed that cells positive for PDGFRβ, CD146, and CD166 exhibited enhanced cell homogeneity with decreased neurogenic subpopulations. These findings support the hypothesis that sorting of hiPSC-derived chondroprogenitor cells with surface markers can be used to purify progenitor cells with enhanced chondrogenic potential, without the need for genetic modification to improve hiPSC chondrogenesis [25, 26].

We previously reported that chondroprogenitor cells at the end of mesodermal lineage differentiation had a high expression of CD146 and CD166 [26]. In the present study, we observed that these markers were also co-expressed with *COL2A1*. CD146 and CD166, along with CD105, have also been shown to be expressed in chondroprogenitors in articular cartilage [19–21]. While our chondroprogenitor cells did not co-express CD105 (ENG) with *COL2A1*, sorting did enrich *CD105* gene expression. Interestingly, it has been shown that CD105 itself may not indicate chondrogenic potential [33]. In addition, scRNA-seq showed that sorted cells exhibited increased expression of *ITGB1* (*CD29*) and *ITGA5* (*CD49e*), which have been deemed necessary for chondrogenic differentiation in progenitor cells and MSCs [18, 19, 34]. Nevertheless, our chondroprogenitor cells had somewhat different expression profiles than skeletal progenitor cells identified previously in vivo [23, 24]. Moderate expression of CD164, a surface marker of the skeletal stem cell [23], was conserved between the unsorted and sorted chondroprogenitor cells while many other markers described were absent from both populations including prechondrocyte markers BMPR1β and CD73 (NT5E) [24]. Therefore, the chondroprogenitor population described in this study is a distinct, unique subpopulation of iPSCs that possesses robust chondrogenic potential.

Several factors may contribute to the differences in cell surface markers that have been identified as markers of chondrogenesis in these different cell types. First, in our study, we used a differentiation protocol which follows the paraxial mesodermal lineage of cartilage [26, 35]. Different types of cartilage follow various developmental pathways (e.g., paraxial mesoderm vs. lateral plate mesoderm), and therefore, the other studies could be investigating these lineages; thus, the cells would have different surface marker expression during differentiation [35–37]. Another explanation may be the time point along the developmental pathway in which the cells are being investigated. Our surface marker profiles are based on the expression of *COL2A1*. While COL2A1 is one of the most prominent matrix proteins in articular cartilage [4] and can indicate chondrogenic potential and determination of a chondrogenic fate [38], *COL2A1* is a relatively late marker of chondrogenesis [39]. Therefore, differences between the cell surface markers identified in our study as compared to other previous work may reflect differences in the prescribed differentiation pathway or the specific subpopulation identified.

In addition to the fact that *COL2A1* expression is a later chondrogenic marker, *COL2A1* expression was found throughout the entire unsorted population including neurogenic cells and sorting significantly decreased its overall expression indicating that *COL2A1*^+^ cells were heterogeneous. This finding is consistent with studies showing that *COL2A1* expression may be a broader indicator for the initial lineage specification of a variety of tissues rather than a sole marker for chondrogenesis during embryonic development [39–41]. Indeed, it has been reported that *COL2A1* is expressed in the floor plate of the central nervous system [42], which provides a plausible explanation for our observation of *COL2A1* expression in neurogenic cells. This may also explain why there are many *COL2A1-*positive cells not expressing the selected surface markers. CD146, CD166, and PDGFRβ may be specific to chondroprogenitors as opposed to cells of other lineages also expressing collagen type II; thus, purifying the population as shown with increased COL2A1 IHC labeling when compared to sorting for *COL2A1* alone. Following sorting for these markers, the size of the chondrogenic *SOX9/COL2A1* population was increased and, while the neural *SOX2* populations were reduced, a *SOX2/TTR* population remained. In fact, this population had a high expression of CD47, an integrin-associated and modulating protein [28] that could be used as an additional marker for sorting in future experiments to improve homogeneity. The expression of nestin and several mesenchyme markers appeared to be permissive in sorted cells, suggesting that PDGFRβ/CD146/CD166 triple-positive cells may still have a similar signature as neural crest cells [43, 44] and might come primarily from mesenchyme populations in unsorted cells. Nonetheless, despite the presence of 6 unique cell clusters, including the *SOX2/TTR* population, sorted chondroprogenitor cells showed robust chondrogenic capacity.

The sorted chondroprogenitors, which all express PDGFRβ, CD146, and CD166, were found to be localized in the mesenchyme clusters of unsorted cells. The alignment of the unsorted and sorted populations by CCA allowed us to compare similarities and differences between the two groups. After alignment, the largest cell cluster expressed histone H4 (*HIST1H4C*). Histones are primarily synthesized during the S-phase of the cell cycle to package the replicated DNA [45], thus indicating the large portion of cells in both sorted and unsorted populations are proliferative. Furthermore, there was a decrease in insulin-like growth factor binding protein-5 (*IGFBP5)* expression in sorted cells among the *IGFBP5/CO2A1* population compared to unsorted. IGFBP5 plays a role in insulin-like growth factor-1 (IGF-1)-dependent chondrocyte proliferation [46] and protects cartilage during OA-induced degeneration [47]. It is possible that sorted cells may be precursors not fully committed to chondrogenic lineage in comparison with unsorted cells. This could be further supported by the observation that sorted cells had increased expression in neural crest and proliferation markers (i.e., *SOX4* and *TUBA1A*, respectively) [48]. Indeed, for all populations identified in the sorted cells, we found that they exhibited elevated expression in proliferative and mesenchymal genes, further suggesting that sorted cells were primarily derived from mesenchyme populations in unsorted cells. Nonetheless, subpopulations in sorted cells still expressed unique gene signatures as shown by the clustering. This finding implies that chondrocytes may differentiate from mesenchyme cells with a variety of transcriptomic profiles if given the correct signaling cues with appropriate timing.

Cartilaginous pellets derived from sorted chondroprogenitor cells showed a significant increase in chondrogenic matrix production and gene expression along with the elimination of a surrounding layer of non-chondrocyte-like cells. Despite the increase in *COL1A1* gene expression, COL1A1 protein, as indicated by IHC labeling, does not reflect its gene expression, implying a potential possibility of post-transcriptional regulation of COL1A1 in protein translation [49]. These results also suggest that the matrix produced by the hiPSC-derived chondrocytes is similar to hyaline cartilage instead of fibrocartilage which is rich in COL1A1 protein. Surprisingly, there was also a relatively small increase in IHC labeling of COL10A1, a matrix protein often associated with hypertrophic chondrocytes [50, 51]. Interestingly, COL6A1 was observed to be more localized around the cells in pellets derived from sorted cells. In developing neonatal cartilage, COL6A1 is found throughout the matrix, but with maturity, it is only found in the pericellular matrix surrounding the chondrocytes [52–54]. The increased expression in COL10A1 at both mRNA and protein levels alongside the co-localization of COL6A1 around chondrocytes suggests that the chondrocytes derived from the sorted cells were at more mature stages as compared to the chondrocytes derived from unsorted cells after 28 days of chondrogenic culture. With maturity and COL10A1 secretion, there is a possibility that these cells may further differentiate into hypertrophic chondrocytes and undergo endochondral ossification. Future studies could be done to investigate the differentiation trajectory with more time in culture and in vivo.

As cell sorting can significantly decrease the number of functional cells [55], we also examined the effects of cell expansion on the differentiation potential of the sorted cells prior to chondrogenesis. Cells in the first passage following sorting exhibit high chondrogenic potential and sGAG staining in pellet culture. However, in subsequent passages, cells showed signs of dedifferentiation and loss of chondrogenic capacity, similar to that observed in primary chondrocytes [56] as well as similarly sorted mouse iPSCs [25]. The decreased chondrogenic potential of sorted cells may result from telomere erosion [57], plating density (e.g., cell-cell and cell-matrix interactions) [56, 58–60], mechanobiological factors (e.g., plate stiffness and/or coating) [61, 62], or culture medium (e.g., low vs. high glucose, growth factors) [58, 61]. While we used an expansion media similar to MSC expansion media due to similarities of the cells, in the future, the media could be altered by changing the glucose level [60] and/or adding growth factors such as fibroblastic growth factor (FGF)-2 and FGF-4, bone morphogenic protein (BMP)-2 and BMP-3, transforming growth factor-beta (TGFβ)-3, heparin-binding epidermal growth factor (EGF), and platelet-derived growth factor (PDGF)-BB [58, 61, 63] as these have been shown to maintain and improve multipotency and chondrogenic capacity.

## Conclusions

In conclusion, we have identified a unique chondroprogenitor population from hiPSCs which expresses PDGFRβ, CD146, and CD166 and has strong chondrogenic potential. While the population does share some characteristics with previously defined chondroprogenitors and traditionally defined MSCs, it has a distinct profile. The methods and findings in this study will contribute to future cartilage tissue engineering and disease modeling studies to improve the understanding and treatment of joint diseases such as OA.

## Supplementary information

**Additional file 1: Figure S1.** related to Figure 2 and 3. GO enrichment analysis of unsorted and sorted cells. (A) Top 10 GO terms (biological process) that were associated with each population in unsorted cells. (B) CD47 was highly expressed in *SOX2/TTR+* cells. (C) Top 10 GO terms (biological process) that were associated with each population in sorted cells. **Figure S2.** related to Figure 2 and 3. Overall gene expression of sorted and unsorted chondroprogenitors. RT-qPCR reveals differences between sorted and unsorted chondroprogenitor cells in overall expression of (A) neurogenic, (B) mesenchymal, and (C) chondrogenic genes. Gene expression in reference to undifferentiated hiPSCs with housekeeping gene TBP. * *p* < 0.05. *** *p* < 0.001. Data represented as mean ± SEM. *n* = 4 samples/group. **Figure S3** related to Figure 2 and 3. Expression profiles of pro-chondrogenic genes in sorted and unsorted chondroprogenitor cells. scRNA-seq reveals that sorted and unsorted cells had distinct gene expression patterns of several markers that were proposed to be pro-chondrogenic identified by previous studies. **Figure S4** related to Fig. 5. Histology for matrix proteins. Safranin-O staining for sGAG showing pellets derived from sorted chondroprogenitor cells had more robust staining and homogenous cell morphology compared to pellets derived from unsorted cells in two individual experimental replicates. Scale bar = 200 μm. Inset scale bar = 400 μm. **Figure S5** related to Figure 5. IHC labeling for COL6A1. There was more distributed labeling for COL6A1 in unsorted chondroprogenitor pellets compared to the localization around cells in sorted chondroprogenitor pellets. Scale bar = 200 μm. Inset scale bar = 400 μm. **Figure S6** related to Figure 6. Alternative analysis of gene expression. Expression of chondrogenic genes *ACAN, SOX9,* and *COL2A1,* fibrocartilage and bone matrix marker *COL1A1,* and hypertrophic cartilage marker *COL10A1* was significantly increased with sorting. C_T_ value of gene of interest was normalized to C_T_ value of housekeeping gene TBP for each sample. ** *p* < 0.01. *** p < 0.001. **** *p* < 0.0001. Data represented as mean ± SEM. *n* = 6-7 per group: 2 experimental replicates, 3-4 technical replicates (pellets). **Figure S7**. Histology of pellets derived from in vitro expanded unsorted and sorted chondroprogenitors. Chondrogenic capacity was maintained after one passage of both unsorted and sorted chondroprogenitor cells as shown by staining for sGAG. There was more robust staining in pellets derived from sorted cells. Safranin-O staining for sGAG showed similar loss of chondrogenic capacity for both unsorted and sorted chondroprogenitor cells through four passages. **Table S1**. Antibodies used for flow cytometry and sorting. **Table S2**. Human primer sequences. Primers were used for RT-qPCR and are listed as 5’ to 3’.

## Data Availability

All sequencing data will be deposited in GEO upon publication of the paper.

## Abbreviations

ASC: Adipose-derived stem cell
BSA: Bovine serum albumin
Cas9: CRISPR-associated protein 9
CCA: Canonical correlation analysis
COL1A1: Collagen type one alpha chain one
COL2A1: Collagen type two alpha chain one
COL6A1: Collagen type six alpha chain one
COL10A1: Collagen type ten alpha chain one
CP: Chondroprogenitor
CRISPR: Clustered regularly interspaced short palindromic repeats
DNA: Deoxyribonucleic acid
FACS: Fluorescent activated cell sorting
bFGF: Basic fibroblastic growth factor
G phase: Gap phase
GFP: Green fluorescent protein
GO: Gene ontology
GTAC: Genome Technology Access Center
hiPSC: Human-induced pluripotent stem cells
IHC: Immunohistochemistry
M phase: Mitotic phase
mRNA: Messenger ribonucleic acid
MSC: Mesenchymal stem cell
OA: Osteoarthritis
p: Passage
PBS: Phosphate-buffered saline
P/S: Penicillin and streptomycin
RNA: Ribonucleic acid
RT-qPCR: Reverse transcription-quantitative polymerase chain reaction
S phase: Synthesis phase
Saf-O: Safranin-O stain
scRNA-seq: Single-cell RNA sequencing
sGAG: Sulfated glycosaminoglycan
TBP: TATA-box-binding protein
TGF-β3: Transforming growth factor-beta three
