# Supplementary figures and images for: Prospective isolation of chondroprogenitors from human iPSCs based on cell surface markers identified using a CRISPR-Cas9-generated reporter
Source: Stem Cell Res Ther. 2020 Feb 18;11:66. doi: 10.1186/s13287-020-01597-8 (PMC7026983; doi:10.1186/s13287-020-01597-8)

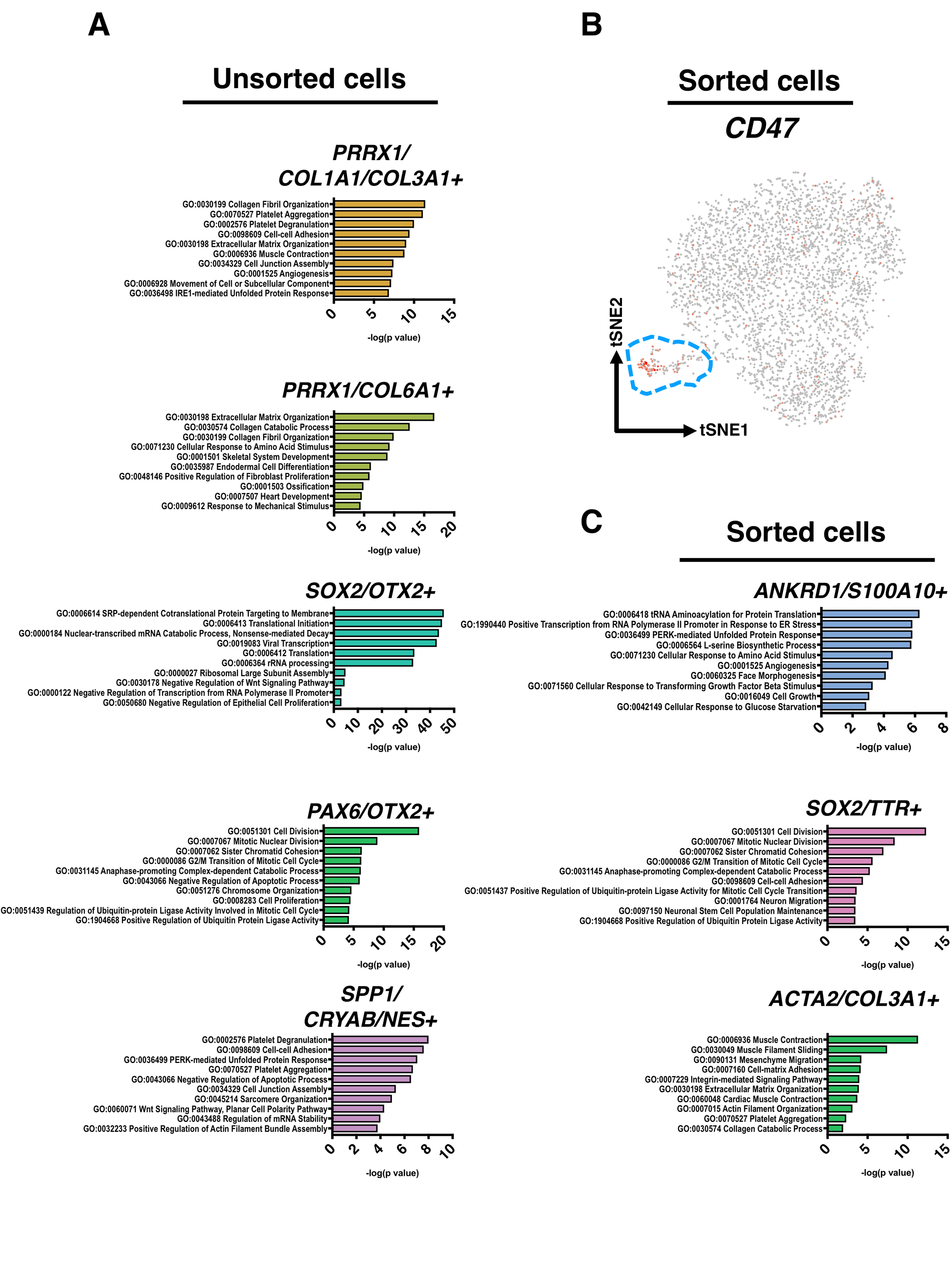

Supplement: Supplementary file 1 — Additional file 1: Figure S1. related to Figure 2 and 3. GO enrichment analysis of unsorted and sorted cells. (A) Top 10 GO terms (biological process) that were associated with each population in unsorted cells. (B) CD47 was highly expressed in SOX2/TTR+ cells. (C) Top 10 GO terms (biological process) that were associated with each population in sorted cells. Figure S2. related to Figure 2 and 3. Overall gene expression of sorted and unsorted chondroprogenitors. RT-qPCR reveals differences between sorted and unsorted chondroprogenitor cells in overall expression of (A) neurogenic, (B) mesenchymal, and (C) chondrogenic genes. Gene expression in reference to undifferentiated hiPSCs with housekeeping gene TBP. * p < 0.05. *** p < 0.001. Data represented as mean ± SEM. n = 4 samples/group. Figure S3 related to Figure 2 and 3. Expression profiles of pro-chondrogenic genes in sorted and unsorted chondroprogenitor cells. scRNA-seq reveals that sorted and unsorted cells had distinct gene expression patterns of several markers that were proposed to be pro-chondrogenic identified by previous studies. Figure S4 related to Fig. 5. Histology for matrix proteins. Safranin-O staining for sGAG showing pellets derived from sorted chondroprogenitor cells had more robust staining and homogenous cell morphology compared to pellets derived from unsorted cells in two individual experimental replicates. Scale bar = 200 μm. Inset scale bar = 400 μm. Figure S5 related to Figure 5. IHC labeling for COL6A1. There was more distributed labeling for COL6A1 in unsorted chondroprogenitor pellets compared to the localization around cells in sorted chondroprogenitor pellets. Scale bar = 200 μm. Inset scale bar = 400 μm. Figure S6 related to Figure 6. Alternative analysis of gene expression. Expression of chondrogenic genes ACAN, SOX9, and COL2A1, fibrocartilage and bone matrix marker COL1A1, and hypertrophic cartilage marker COL10A1 was significantly increased with sorting. CT value of gene of inter [file 13287_2020_1597_MOESM1_ESM.zip › FigS1.tif]

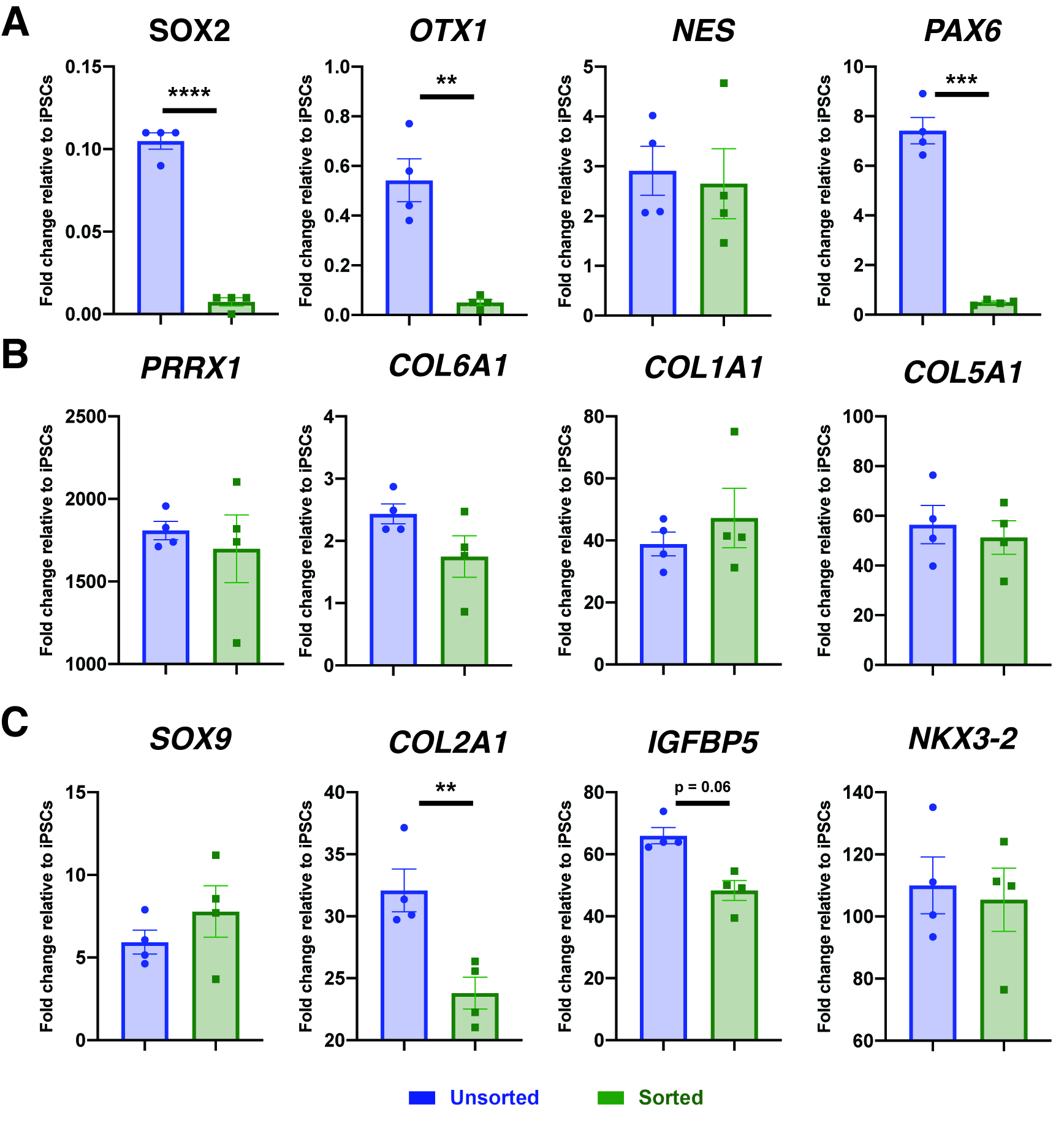

Supplement: Supplementary file 1 — Additional file 1: Figure S1. related to Figure 2 and 3. GO enrichment analysis of unsorted and sorted cells. (A) Top 10 GO terms (biological process) that were associated with each population in unsorted cells. (B) CD47 was highly expressed in SOX2/TTR+ cells. (C) Top 10 GO terms (biological process) that were associated with each population in sorted cells. Figure S2. related to Figure 2 and 3. Overall gene expression of sorted and unsorted chondroprogenitors. RT-qPCR reveals differences between sorted and unsorted chondroprogenitor cells in overall expression of (A) neurogenic, (B) mesenchymal, and (C) chondrogenic genes. Gene expression in reference to undifferentiated hiPSCs with housekeeping gene TBP. * p < 0.05. *** p < 0.001. Data represented as mean ± SEM. n = 4 samples/group. Figure S3 related to Figure 2 and 3. Expression profiles of pro-chondrogenic genes in sorted and unsorted chondroprogenitor cells. scRNA-seq reveals that sorted and unsorted cells had distinct gene expression patterns of several markers that were proposed to be pro-chondrogenic identified by previous studies. Figure S4 related to Fig. 5. Histology for matrix proteins. Safranin-O staining for sGAG showing pellets derived from sorted chondroprogenitor cells had more robust staining and homogenous cell morphology compared to pellets derived from unsorted cells in two individual experimental replicates. Scale bar = 200 μm. Inset scale bar = 400 μm. Figure S5 related to Figure 5. IHC labeling for COL6A1. There was more distributed labeling for COL6A1 in unsorted chondroprogenitor pellets compared to the localization around cells in sorted chondroprogenitor pellets. Scale bar = 200 μm. Inset scale bar = 400 μm. Figure S6 related to Figure 6. Alternative analysis of gene expression. Expression of chondrogenic genes ACAN, SOX9, and COL2A1, fibrocartilage and bone matrix marker COL1A1, and hypertrophic cartilage marker COL10A1 was significantly increased with sorting. CT value of gene of inter [file 13287_2020_1597_MOESM1_ESM.zip › FigS2.tif]

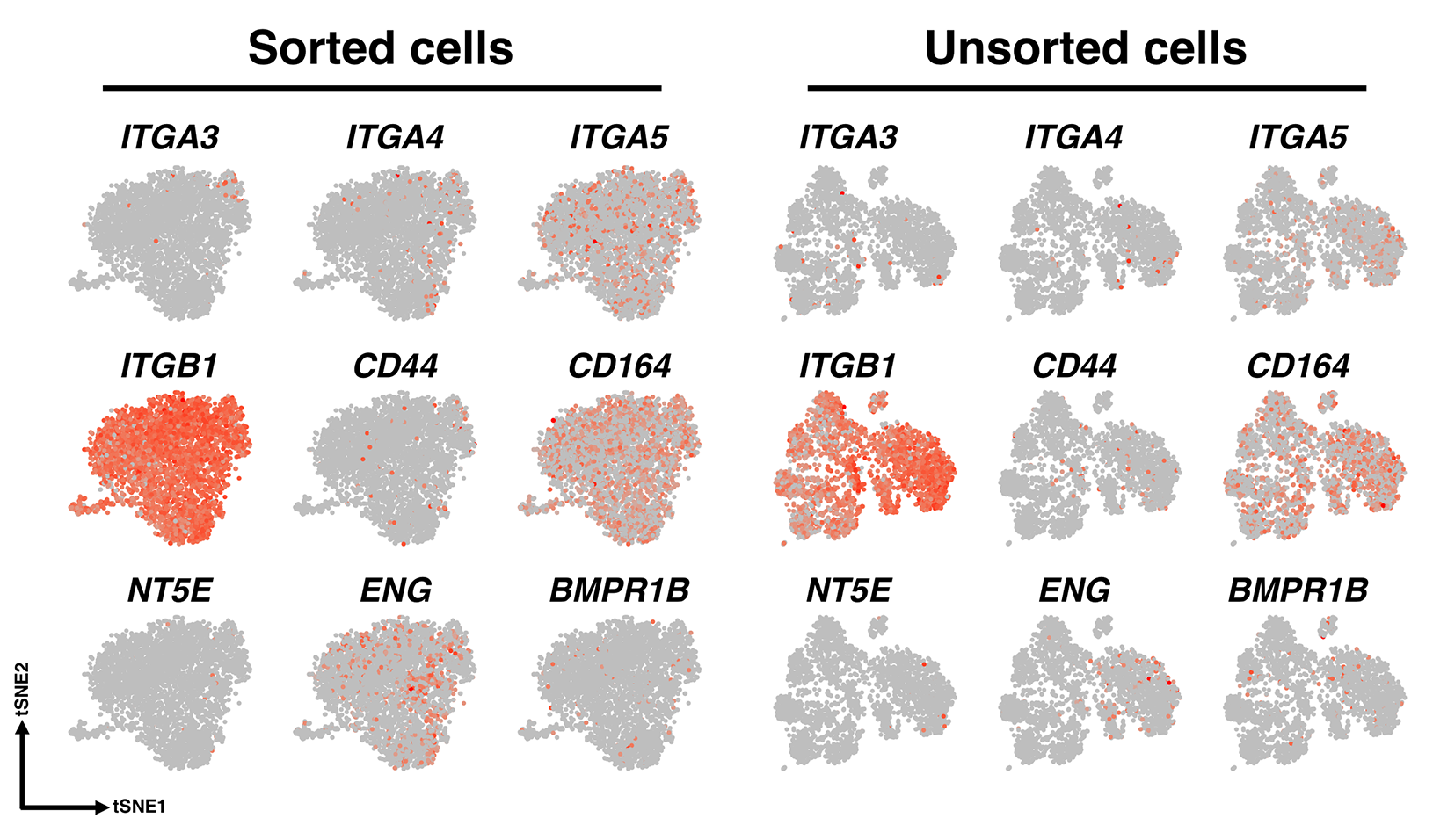

Supplement: Supplementary file 1 — Additional file 1: Figure S1. related to Figure 2 and 3. GO enrichment analysis of unsorted and sorted cells. (A) Top 10 GO terms (biological process) that were associated with each population in unsorted cells. (B) CD47 was highly expressed in SOX2/TTR+ cells. (C) Top 10 GO terms (biological process) that were associated with each population in sorted cells. Figure S2. related to Figure 2 and 3. Overall gene expression of sorted and unsorted chondroprogenitors. RT-qPCR reveals differences between sorted and unsorted chondroprogenitor cells in overall expression of (A) neurogenic, (B) mesenchymal, and (C) chondrogenic genes. Gene expression in reference to undifferentiated hiPSCs with housekeeping gene TBP. * p < 0.05. *** p < 0.001. Data represented as mean ± SEM. n = 4 samples/group. Figure S3 related to Figure 2 and 3. Expression profiles of pro-chondrogenic genes in sorted and unsorted chondroprogenitor cells. scRNA-seq reveals that sorted and unsorted cells had distinct gene expression patterns of several markers that were proposed to be pro-chondrogenic identified by previous studies. Figure S4 related to Fig. 5. Histology for matrix proteins. Safranin-O staining for sGAG showing pellets derived from sorted chondroprogenitor cells had more robust staining and homogenous cell morphology compared to pellets derived from unsorted cells in two individual experimental replicates. Scale bar = 200 μm. Inset scale bar = 400 μm. Figure S5 related to Figure 5. IHC labeling for COL6A1. There was more distributed labeling for COL6A1 in unsorted chondroprogenitor pellets compared to the localization around cells in sorted chondroprogenitor pellets. Scale bar = 200 μm. Inset scale bar = 400 μm. Figure S6 related to Figure 6. Alternative analysis of gene expression. Expression of chondrogenic genes ACAN, SOX9, and COL2A1, fibrocartilage and bone matrix marker COL1A1, and hypertrophic cartilage marker COL10A1 was significantly increased with sorting. CT value of gene of inter [file 13287_2020_1597_MOESM1_ESM.zip › FigS3.tif]

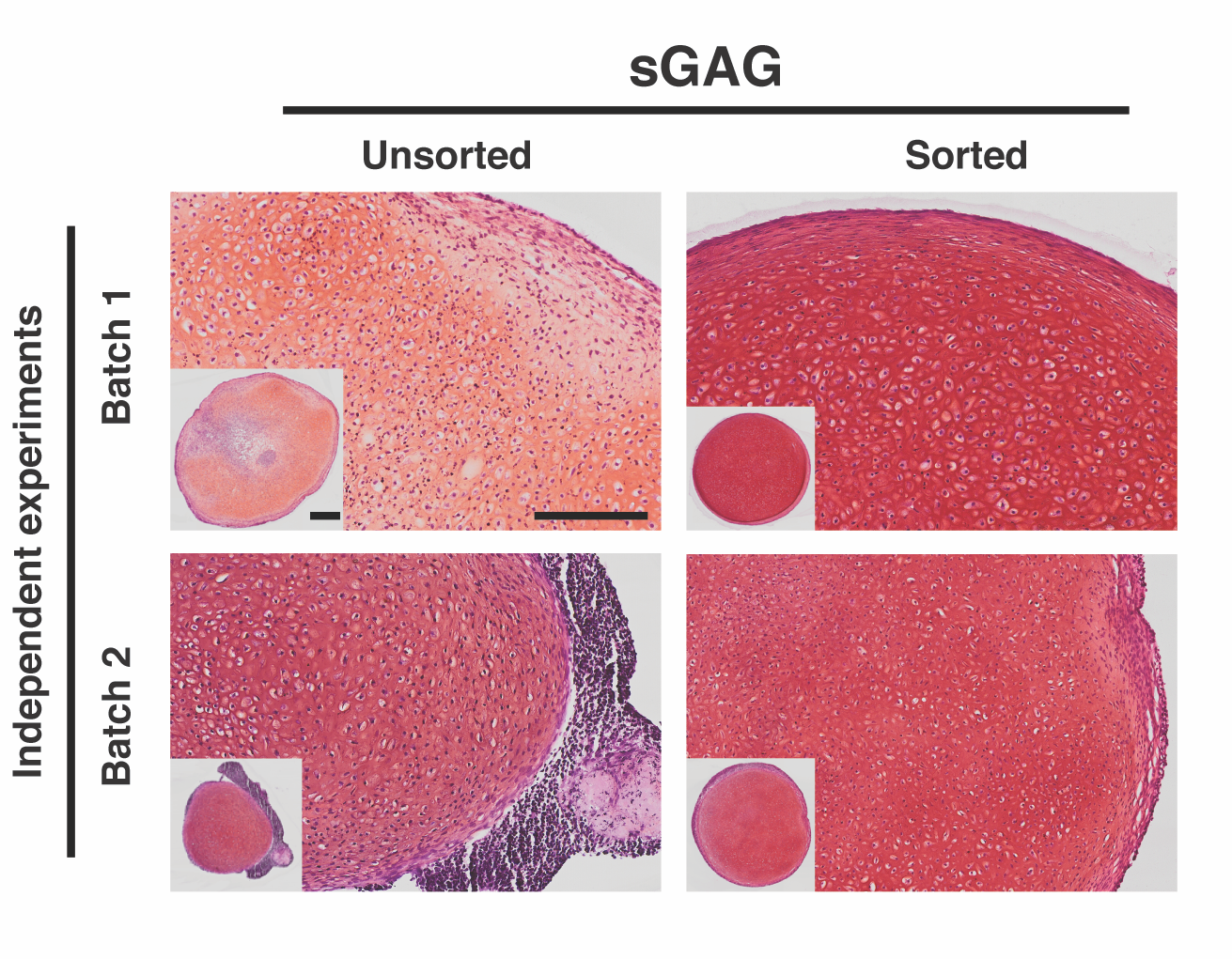

Supplement: Supplementary file 1 — Additional file 1: Figure S1. related to Figure 2 and 3. GO enrichment analysis of unsorted and sorted cells. (A) Top 10 GO terms (biological process) that were associated with each population in unsorted cells. (B) CD47 was highly expressed in SOX2/TTR+ cells. (C) Top 10 GO terms (biological process) that were associated with each population in sorted cells. Figure S2. related to Figure 2 and 3. Overall gene expression of sorted and unsorted chondroprogenitors. RT-qPCR reveals differences between sorted and unsorted chondroprogenitor cells in overall expression of (A) neurogenic, (B) mesenchymal, and (C) chondrogenic genes. Gene expression in reference to undifferentiated hiPSCs with housekeeping gene TBP. * p < 0.05. *** p < 0.001. Data represented as mean ± SEM. n = 4 samples/group. Figure S3 related to Figure 2 and 3. Expression profiles of pro-chondrogenic genes in sorted and unsorted chondroprogenitor cells. scRNA-seq reveals that sorted and unsorted cells had distinct gene expression patterns of several markers that were proposed to be pro-chondrogenic identified by previous studies. Figure S4 related to Fig. 5. Histology for matrix proteins. Safranin-O staining for sGAG showing pellets derived from sorted chondroprogenitor cells had more robust staining and homogenous cell morphology compared to pellets derived from unsorted cells in two individual experimental replicates. Scale bar = 200 μm. Inset scale bar = 400 μm. Figure S5 related to Figure 5. IHC labeling for COL6A1. There was more distributed labeling for COL6A1 in unsorted chondroprogenitor pellets compared to the localization around cells in sorted chondroprogenitor pellets. Scale bar = 200 μm. Inset scale bar = 400 μm. Figure S6 related to Figure 6. Alternative analysis of gene expression. Expression of chondrogenic genes ACAN, SOX9, and COL2A1, fibrocartilage and bone matrix marker COL1A1, and hypertrophic cartilage marker COL10A1 was significantly increased with sorting. CT value of gene of inter [file 13287_2020_1597_MOESM1_ESM.zip › FigS4.tif]

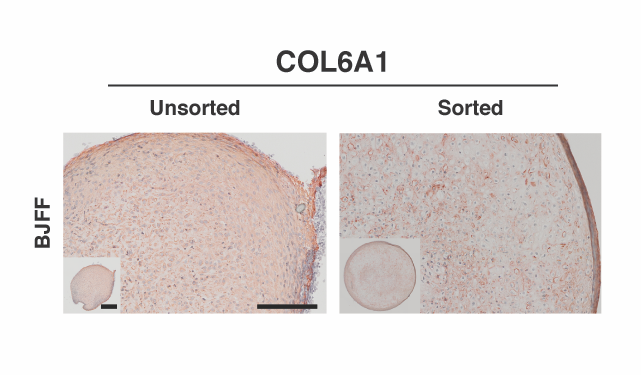

Supplement: Supplementary file 1 — Additional file 1: Figure S1. related to Figure 2 and 3. GO enrichment analysis of unsorted and sorted cells. (A) Top 10 GO terms (biological process) that were associated with each population in unsorted cells. (B) CD47 was highly expressed in SOX2/TTR+ cells. (C) Top 10 GO terms (biological process) that were associated with each population in sorted cells. Figure S2. related to Figure 2 and 3. Overall gene expression of sorted and unsorted chondroprogenitors. RT-qPCR reveals differences between sorted and unsorted chondroprogenitor cells in overall expression of (A) neurogenic, (B) mesenchymal, and (C) chondrogenic genes. Gene expression in reference to undifferentiated hiPSCs with housekeeping gene TBP. * p < 0.05. *** p < 0.001. Data represented as mean ± SEM. n = 4 samples/group. Figure S3 related to Figure 2 and 3. Expression profiles of pro-chondrogenic genes in sorted and unsorted chondroprogenitor cells. scRNA-seq reveals that sorted and unsorted cells had distinct gene expression patterns of several markers that were proposed to be pro-chondrogenic identified by previous studies. Figure S4 related to Fig. 5. Histology for matrix proteins. Safranin-O staining for sGAG showing pellets derived from sorted chondroprogenitor cells had more robust staining and homogenous cell morphology compared to pellets derived from unsorted cells in two individual experimental replicates. Scale bar = 200 μm. Inset scale bar = 400 μm. Figure S5 related to Figure 5. IHC labeling for COL6A1. There was more distributed labeling for COL6A1 in unsorted chondroprogenitor pellets compared to the localization around cells in sorted chondroprogenitor pellets. Scale bar = 200 μm. Inset scale bar = 400 μm. Figure S6 related to Figure 6. Alternative analysis of gene expression. Expression of chondrogenic genes ACAN, SOX9, and COL2A1, fibrocartilage and bone matrix marker COL1A1, and hypertrophic cartilage marker COL10A1 was significantly increased with sorting. CT value of gene of inter [file 13287_2020_1597_MOESM1_ESM.zip › FigS5.tif]

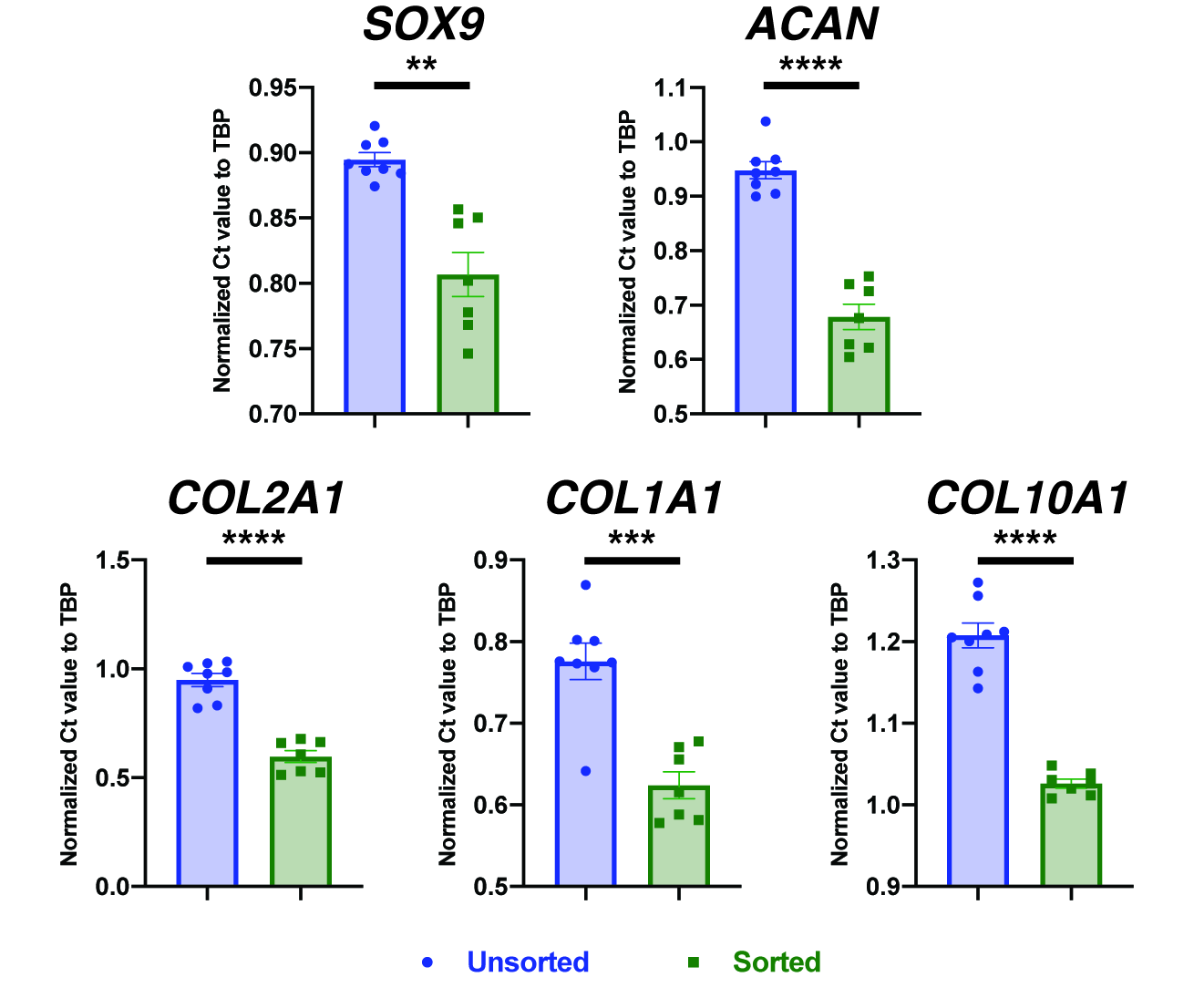

Supplement: Supplementary file 1 — Additional file 1: Figure S1. related to Figure 2 and 3. GO enrichment analysis of unsorted and sorted cells. (A) Top 10 GO terms (biological process) that were associated with each population in unsorted cells. (B) CD47 was highly expressed in SOX2/TTR+ cells. (C) Top 10 GO terms (biological process) that were associated with each population in sorted cells. Figure S2. related to Figure 2 and 3. Overall gene expression of sorted and unsorted chondroprogenitors. RT-qPCR reveals differences between sorted and unsorted chondroprogenitor cells in overall expression of (A) neurogenic, (B) mesenchymal, and (C) chondrogenic genes. Gene expression in reference to undifferentiated hiPSCs with housekeeping gene TBP. * p < 0.05. *** p < 0.001. Data represented as mean ± SEM. n = 4 samples/group. Figure S3 related to Figure 2 and 3. Expression profiles of pro-chondrogenic genes in sorted and unsorted chondroprogenitor cells. scRNA-seq reveals that sorted and unsorted cells had distinct gene expression patterns of several markers that were proposed to be pro-chondrogenic identified by previous studies. Figure S4 related to Fig. 5. Histology for matrix proteins. Safranin-O staining for sGAG showing pellets derived from sorted chondroprogenitor cells had more robust staining and homogenous cell morphology compared to pellets derived from unsorted cells in two individual experimental replicates. Scale bar = 200 μm. Inset scale bar = 400 μm. Figure S5 related to Figure 5. IHC labeling for COL6A1. There was more distributed labeling for COL6A1 in unsorted chondroprogenitor pellets compared to the localization around cells in sorted chondroprogenitor pellets. Scale bar = 200 μm. Inset scale bar = 400 μm. Figure S6 related to Figure 6. Alternative analysis of gene expression. Expression of chondrogenic genes ACAN, SOX9, and COL2A1, fibrocartilage and bone matrix marker COL1A1, and hypertrophic cartilage marker COL10A1 was significantly increased with sorting. CT value of gene of inter [file 13287_2020_1597_MOESM1_ESM.zip › FigS6.tif]

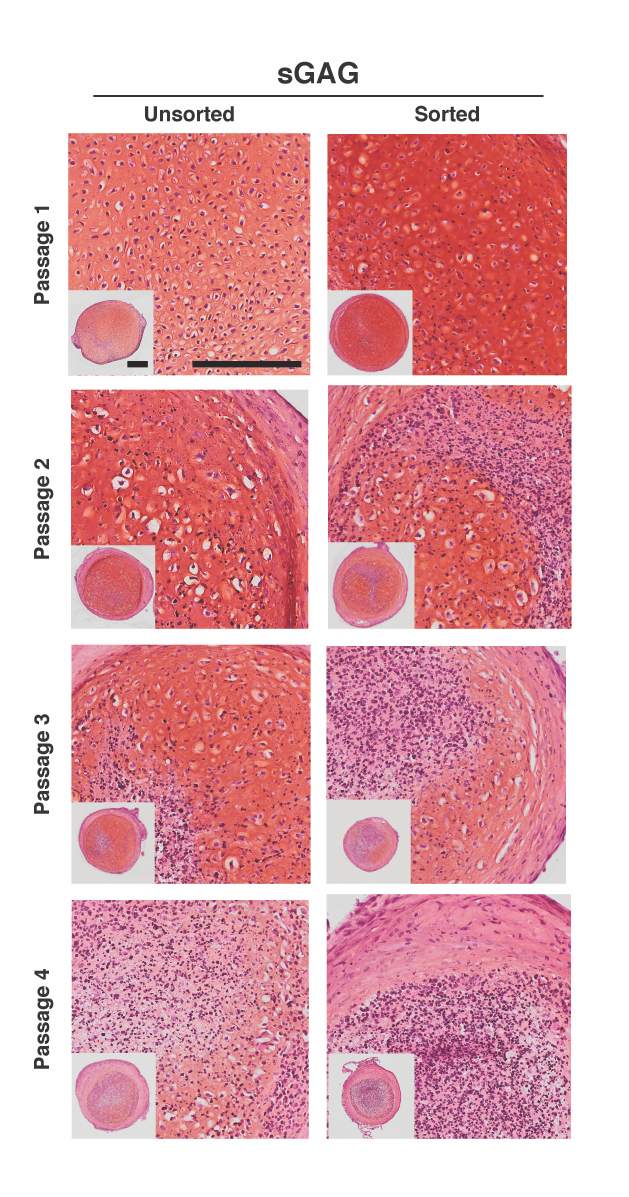

Supplement: Supplementary file 1 — Additional file 1: Figure S1. related to Figure 2 and 3. GO enrichment analysis of unsorted and sorted cells. (A) Top 10 GO terms (biological process) that were associated with each population in unsorted cells. (B) CD47 was highly expressed in SOX2/TTR+ cells. (C) Top 10 GO terms (biological process) that were associated with each population in sorted cells. Figure S2. related to Figure 2 and 3. Overall gene expression of sorted and unsorted chondroprogenitors. RT-qPCR reveals differences between sorted and unsorted chondroprogenitor cells in overall expression of (A) neurogenic, (B) mesenchymal, and (C) chondrogenic genes. Gene expression in reference to undifferentiated hiPSCs with housekeeping gene TBP. * p < 0.05. *** p < 0.001. Data represented as mean ± SEM. n = 4 samples/group. Figure S3 related to Figure 2 and 3. Expression profiles of pro-chondrogenic genes in sorted and unsorted chondroprogenitor cells. scRNA-seq reveals that sorted and unsorted cells had distinct gene expression patterns of several markers that were proposed to be pro-chondrogenic identified by previous studies. Figure S4 related to Fig. 5. Histology for matrix proteins. Safranin-O staining for sGAG showing pellets derived from sorted chondroprogenitor cells had more robust staining and homogenous cell morphology compared to pellets derived from unsorted cells in two individual experimental replicates. Scale bar = 200 μm. Inset scale bar = 400 μm. Figure S5 related to Figure 5. IHC labeling for COL6A1. There was more distributed labeling for COL6A1 in unsorted chondroprogenitor pellets compared to the localization around cells in sorted chondroprogenitor pellets. Scale bar = 200 μm. Inset scale bar = 400 μm. Figure S6 related to Figure 6. Alternative analysis of gene expression. Expression of chondrogenic genes ACAN, SOX9, and COL2A1, fibrocartilage and bone matrix marker COL1A1, and hypertrophic cartilage marker COL10A1 was significantly increased with sorting. CT value of gene of inter [file 13287_2020_1597_MOESM1_ESM.zip › FigS7.tif]
